# Aristaless-Related Homeobox Plays a Key Role in Hyperplasia of the Pancreas Islet α–Like Cells in Mice Deficient in Proglucagon-Derived Peptides

**DOI:** 10.1371/journal.pone.0064415

**Published:** 2013-05-09

**Authors:** Sai Xu, Yoshitaka Hayashi, Yoshiko Takagishi, Mariko Itoh, Yoshiharu Murata

**Affiliations:** 1 Department of Genetics, Division of Stress Adaptation and Protection, Research Institute of Environmental Medicine, Nagoya University, Nagoya, Japan; 2 Technical Department, Research Institute of Environmental Medicine, Nagoya University, Nagoya, Japan; University of Bremen, Germany

## Abstract

Defects in glucagon action can cause hyperplasia of islet α-cells, however, the underlying mechanisms remain largely to be elucidated. Mice homozygous for a glucagon-GFP knock-in allele (*Gcg^gfp/gfp^*) completely lack proglucagon-derived peptides and exhibit hyperplasia of GFP-positive α-like cells. Expression of the transcription factor, aristaless-related homeobox (ARX), is also increased in the *Gcg^gfp/gfp^* pancreas. Here, we sought to elucidate the role of ARX in the hyperplasia of α-like cells through analyses of two *Arx* mutant alleles (*Arx^P355L/Y^* and *Arx^ [330insGCG]7/Y^*) that have different levels of impairment of their function. Expression of *Gfp* and *Arx* genes was higher and the size and number of islets increased in the *Gcg^gfp/gfp^* pancreas compared to and *Gcg^gfp/+^* pancreas at 2 weeks of age. In male *Gcg^gfp/gfp^* mice that are hemizygous for the *Arx^P355L/Y^* mutation that results in a protein with a P355L amino acid substitution, expression of *Gfp* mRNA in the pancreas was comparable to that in control *Gcg^gfp/+^Arx^+/Y^* mice. The increases in islet size and number were also reduced in these mice. Immunohistochemical analysis showed that the number of GFP-positive cells was comparable in *Gcg^gfp/gfp^ Arx^P355L/Y^* and *Gcg^gfp/+^Arx^+/Y^* mice. These results indicate that the hyperplasia is reduced by introduction of an *Arx* mutation. *Arx^P355L/Y^* mice appeared to be phenotypically normal; however, *Arx^ [330insGCG]7/Y^* mice that have a mutant ARX protein with expansion of the polyalanine tract had a reduced body size and shortened life span. The number of GFP positive cells was further reduced in the *Gcg^gfp/gfp^ Arx^ [330insGCG]7/Y^* mice. Taken together, our findings show that the function of ARX is one of the key modifiers for hyperplasia of islet α-like cells in the absence of proglucagon-derived peptides.

## Introduction

Multiple bioactive peptides, including glucagon and glucagon-like peptides (GLPs) are produced through cell-type specific cleavage of proglucagon, which is encoded by the glucagon gene (*Gcg*) [1], [2], [3]. In order to gain insights into the physiological function of proglucagon-derived peptides, we recently generated *Gcg*-GFP (green fluorescent protein) knock-in mice (*Gcg^gfp/+^*). Homozygous *Gcg^gfp/gfp^* mice lack all proglucagon-derived peptides and develop prominent hyperplasia of islet α-like cells, which are GFP-positive but do not contain glucagon; by contrast, hyperplasia of intestinal L-like cells, which are also GFP-positive but do not contain GLPs, was not observed [4]. We also found that hyperplasia of α-like cells was associated with a marked increase in the level of *Aristaless-related homeobox* (*Arx*) mRNA [4].

*Arx* is a homeobox gene that is expressed in the central nervous system and plays an important role in brain development [5]. The gene is located on the X-chromosome in both humans and mice, and mutations of the gene cause severe X-linked neurological disorders in humans [6], [7]. ARX also plays a pivotal role in the development of pancreatic islet α-cells; *Arx*-null mice fail to develop mature islet α-cells with a concomitant increase in the numbers of ß- and ∂-cells [8]. Conversely, forced expression of ARX in mature ß-cells promotes a conversion of the cells into glucagon-producing cells [9]. The present study was aimed to characterize the role of Arx in the hyperplasia of GFP-positive, α-like cells in the pancreatic islets of *Gcg^gfp/gfp^* mice.

*Arx* null mice die at 2 days after birth [8], and therefore cannot be used for analyzing postnatal hyperplasia of α-like cells in the *Gcg^gfp/gfp^* mice [4]. To avoid this difficulty, we obtained two mouse strains with partial defects in ARX functions: one strain has elongation of GCG-triplet repeats, which encode the polyalanine tract (330ins[GCG]7); the other strain has an amino acid substitution (P355L, equivalent to the amino acid number 353 in human) [10]. Functional impairment of ARX-330ins [GCG]7 is more severe than ARX-P355/353L, as the *Arx^ [330insGCG]7/Y^* mice exhibit greater neurological abnormalities than *Arx^P355L/Y^* mice [10]. Homologous human mutations have been identified in patients with X-linked mental retardation: a homologue of *Arx*-330ins[GCG]7, hereafter referred to as *Arx*-7, was identified in patients with X-linked infantile spasms syndrome/West syndrome, and a homologue of *Arx*-P355/353L, hereafter referred to as *Arx*-PL, was found in patients with X-linked myoclonic epilepsy with generalized spasticity and intellectual disability [10]. Through crosses between *Arx* mutant mice and *Gcg^gfp/gfp^* mice, we generated 6 different male mutant genotypes: *Gcg^gfp/+^Arx^+/Y^*, *Gcg^gfp/+^Arx^PL/Y^*, *Gcg^gfp/+^Arx^7/Y^*, *Gcg^gfp/gfp^Arx^+/Y^*, *Gcg^gfp/gfp^Arx^PL/Y^* and *Gcg^gfp/gfp^Arx^7/Y^*. These mice were used in the analyses described below.

## Materials and Methods

### Animals

We generated heterozygous Glucagon-GFP knock-in (*Gcg^gfp/+^*) mice as described previously and backcrossed the mice to the C57BL/6J strain for more than 10 generations [11]. Females carrying an *Arx* mutation (*Arx^PL/X^* or *Arx^7/X^*) on a C57BL/6J genetic background [10] were obtained from the Experimental Animal Division, RIKEN Bioresource Center and mated with male *Gcg^gfp/gfp^* mice. Arx/Glucagon double heterozygous females, *Gcg^gfp/+^Arx^7/X^* and *Gcg^gfp/+^Arx^PL/X^*, were then mated with male *Gcg^gfp/gfp^* mice to obtain 6 different male *Gcg/Arx* genotypes: *Gcg^gfp/+^Arx^+/Y^*, *Gcg^gfp/+^Arx^PL/Y^*, *Gcg^gfp/+^Arx^7/Y^*, *Gcg^gfp/gfp^Arx^+/Y^*, *Gcg^gfp/gfp^Arx^PL/Y^* and *Gcg^gfp/gfp^Arx^7/Y^*. All mice were housed in specific pathogen-free (SPF) barrier facilities in the Research Institute of Environmental Medicine, Nagoya University, and maintained on a 12-h light, 12-h dark cycle and constant temperature (23°C) with free access to certified chow (Lab Animal Diet MF; Oriental Yeast Co. Ltd., Tokyo, Japan) and distilled water. This study was carried out in strict accordance with the recommendations in the Guide for the Care and Use of Laboratory Animals of the National Institute of Health. The protocol was approved by the Institutional Animal Care and Use Committee of Research Institute of Environmental Medicine, Nagoya University (Permit numbers: #12114). All efforts were made to minimize suffering of animals.

### Genotyping

DNA was extracted from cells or tail tips by proteinase K digestion and phenol/chloroform extraction. Genotypes were determined by PCR as previously described in detail [4], [10].

### Measurement of blood glucose levels and insulin tolerance test (ITT)

Blood samples were obtained from neck blood vessels and from tail veins in two weeks old and four months old male mice, respectively. Glucose levels were determined using a Medisafe glucose meter (TERUMO, Tokyo, Japan). For the ITT, mice were starved for 4 h and then injected intraperitoneally with porcine insulin (0.25 units/kg, Sigma-Aldrich Japan, Tokyo, Japan). Tail blood samples were taken at 0, 30, 60, 90 and 120 minutes after the injection and blood glucose levels were determined.

### Fluorescent imaging

Two-week-old mice were killed by cervical dislocation. They were immediately dissected to expose the viscera including the pancreas. Fluorescence images, with exposure for 1 or 10 seconds, were captured using a VB-G25 epifluorescence microscope system (Keyence Corp., Osaka, Japan).

### RNA extraction and analysis of gene expression

Total RNA was extracted from the pancreas using an RNeasy mini kit (QIAGEN, Germantown, MD) according to the manufacturer's instructions and cDNAs (cDNAs) were synthesized using random primers. cDNA aliquots equivalent to 1 μg of total RNA were subjected to quantitative real-time PCR. The sequences of the primers used for the analyses are available upon request. Details of the procedure have been described previously [12].

### Histological and quantitative analyses on the pancreatic islets

Each pancreas was dissected, weighed, fixed in 4% paraformaldehyde, and then, embedded in paraffin. The islet area was determined using slight modifications of the previously described method [13], [14], [15]. In brief, the complete pancreas in the paraffin block was cut into 6 µm sections. Sections at 90 µm intervals were stained with hematoxylin and eosin (HE staining) and images of the pancreas were obtained and the dimension/area of the islets was measured using a Nanozoomer 2.0 RS whole slide scanner (Hamamatsu Photonics, Hamamatsu City, Japan). The total pancreatic area was measured using Image Pro Plus 6.1 software (Media Cybernetics, Silver Springs, MD). Islet area was expressed as a proportion (%) of total of the pancreatic area and the density of islets was expressed as per mm^2^. Representative images of HE stained sections were captured using an Olympus BX53 system (Olympus Corporation, Tokyo, Japan).

### Antibodies

The anti-glucagon and insulin antibodies were purchased from Abcam, plc., (Cambridge, UK) and the anti-GFP from Medical & Biological Laboratories Co., LTD. (Nagoya, Japan). The HRP-conjugated secondary antibodies were purchased from Jackson ImmunoResearch (West Grove, PA, USA).

### Statistical analyses

Data are expressed as the mean ± SEM. Statistical analysis was performed using a one-way ANOVA followed by Scheffe's test, using IBM SPSS Statistics software Version 16.0. *P*-values less than 0.05 were regarded as statistically significant.

## Results

### Ontogenetic expression of transcription factors in the pancreas of *Gcg^gfp/gfp^* mice

**Figure 1:**
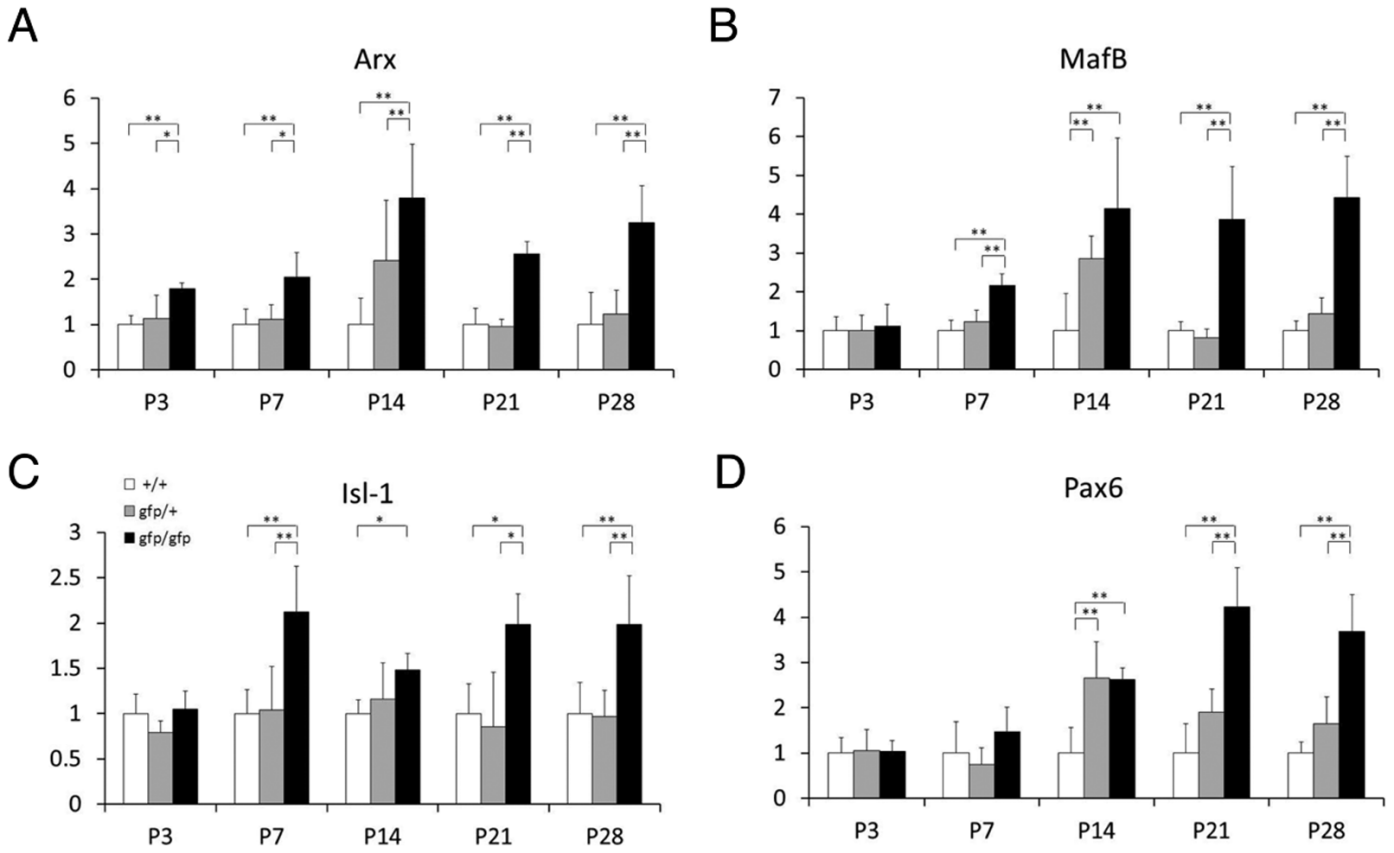
Ontogenetic expression of transcription factors in *Gcg^gfp/gfp^* mice. Relative expression levels of *Arx* (A), *MafB* (B), *Isl-1* (C), and *Pax6* (D) from P3 to P28 are shown as mean ± SEM (N = 5). Open bars, wild-type mice (*Gcg^+/+^*); shaded bars, heterozygous mice (*Gcg^gfp/+^*); closed bars, homozygous mice (*Gcg^gfp/gfp^*). *: P<0.05, **: P<0.01.

The levels of expression of several transcription factors involved in the differentiation of islet endocrine cells were previously shown to be significantly increased in *Gcg^gfp/gfp^* mice, which develop hyperplasia of GFP-positive α-like cells [3]. To characterize the onset of this altered gene expression pattern, we quantified the levels of transcription factor mRNAs during ontogenesis. As shown in Fig. 1A, significantly higher levels of *Arx* mRNA were present at postnatal day 3 (P3) in the *Gcg^gfp/gfp^* pancreas compared to either *Gcg^+/+^* or *Gcg^gfp/+^*. By contrast, similar inter-genotype differences in the levels of *MafB*, *Isl-1*, and *Pax6* mRNAs were not observed until P7 or later. Therefore, the higher level of expression of *Arx* precedes that of the other transcription factor genes analyzed here. This finding suggests that ARX plays a key role in the development of hyperplasia of α-like cells in the *Gcg^gfp/gfp^* pancreas.

### Body weights and blood glucose levels in *Glucagon/Arx* double mutant mice

**Figure 2:**
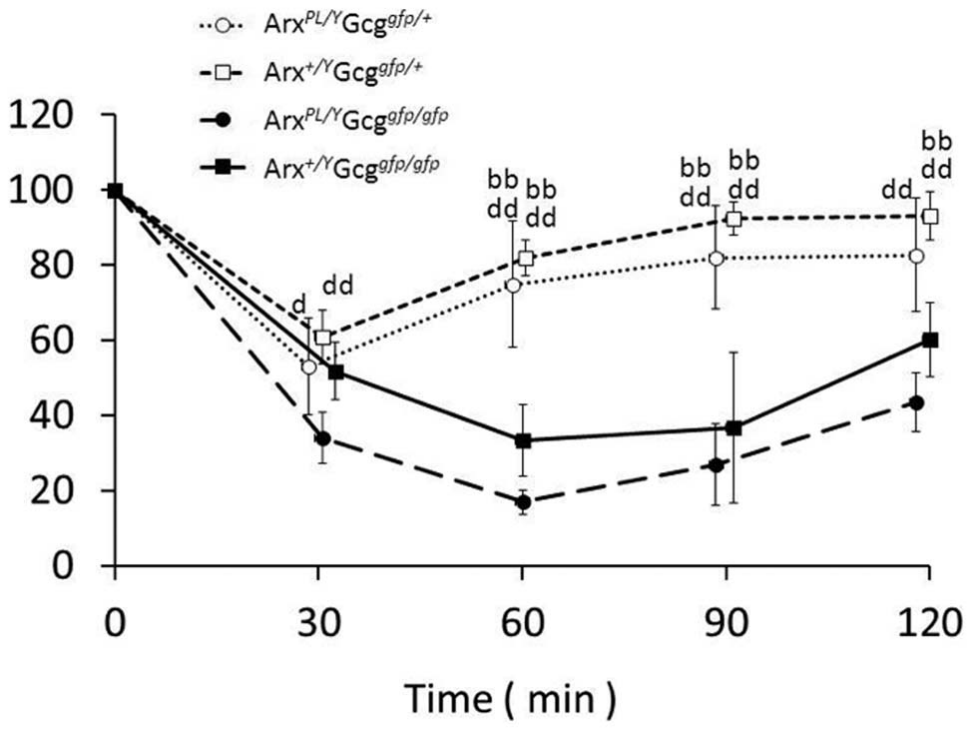
Insulin tolerance tests in glucagon/Arx-PL double mutant mice. Insulin tolerance test was performed using *Gcg^gfp/+^Arx^+/Y^*, *Gcg^gfp/+^Arx^PL/Y^*, *Gcg^gfp/gfp^Arx^+/Y^* and *Gcg^gfp/gfp^Arx^PL/Y^* mutant mice. Open circles, *Gcg^gfp/+^Arx^+/Y^*; open squares, *Gcg^gfp/+^Arx^PL/Y^*; closed circles, *Gcg^gfp/gfp^Arx^+/Y^*; closed squares, *Gcg^gfp/gfp^Arx^PL/Y^*. ‘bb’ vs *Gcg^gfp/gfp^Arx^+/Y^*, P<0.01; ‘d’ vs *Gcg^gfp/gfp^Arx^PL/Y^*, P<0.05; ‘dd’ vs *Gcg^gfp/gfp^Arx^PL/Y^*, P<0.01.

To address the role of ARX in the hyperplasia of α-like cells, we generated *Glucagon/Arx* (*Gcg/Arx*) double mutant mice. We crossed *Gcg^gfp/gfp^* males with females heterozygous for the *Arx* mutation and obtained *Gcg/Arx* double mutant mice in the expected Mendelian ratio. At 2 weeks of age, the body weights of *Arx* ^7/Y^, combined with either *Gcg^gfp/+^* or *Gcg* ^g*fp/gfp*^ were significantly smaller than control *Gcg^gfp/+^Arx^+/Y^* mice (Table 1). The difference in body weights between *Gcg^gfp/gfp^Arx^+/Y^* and *Gcg^gfp/gfp^Arx^7/Y^* mice was also statistically significant. However, the body weights of *Gcg^gfp/+^Arx^PL/Y^* mice were comparable to the control. Blood glucose levels in *Gcg^gfp/gfp^Arx^7/Y^* mice were lower than in *Gcg^gfp/gfp^Arx^X/Y^* mice. These findings indicate that the functional impairment associated with ARX-7 is more severe than for ARX-PL; this conclusion is in concordance with the difference in severity of neurological phenotypes between these *Arx* mutant mice [10]. Furthermore, most of the *Arx^7/Y^* mice died within 3 months, while *Arx^PL/Y^* mice survived for more than 6 months. At 4 months of age, no significant differences in blood glucose levels were detected among *Gcg^gfp/+^Arx^+/Y^*, *Gcg^gfp/gfp^Arx^+/Y^*, *Gcg^gfp/+^Arx^PL/Y^*, and *Gcg^gfp/gfp^Arx^PL/Y^* mice. On insulin loading, the changes in blood glucose levels in the *Gcg^gfp/+^Arx^PL/Y^* mice were comparable to those in *Gcg^gfp/+^Arx^+/Y^* mice (Fig. 2). Taken together, these findings indicate that impairment in growth and blood glucose level control is marginal in the *Arx^PL/Y^* mice, but is more severe in the *Arx^7/Y^* mice.

**Table 1:** Phenotype of the mutant mice.

	*Gcg^gfp/+^Arx^+/Y^*	*Gcg^gfp/gfp^Arx^+/Y^*	*Gcg^gfp/+^Arx^PL/Y^*	*Gcg^gfp/gfp^Arx^PL/Y^*	*Gcg^gfp/+^Arx^7/Y^*	*Gcg^gfp/gfp^Arx^7/Y^*
Life span	>6 months	>6 months	>6 months	>6 months	3–4months	3–4months
Body Weight	6.56±0.45^(d, ee, ff)^	5.75±0.90 ^(f)^	6.27±0.75^(ff)^	5.19±0.40	5.17±0.32	4.46±0.21

(2w old)	(n = 5)	(n = 8)	(n = 5)	(n = 7)	(n = 9)	(n = 5)
Body Weight	28.73±0.64	29.12±1.49	30.0±2.0	27.71±1.42	No data	No data

(4m old)	(n = 5)	(n = 5)	(n = 5)	(n = 3)		
Blood Glucose	107±29	104±50^(f)^	107±25	66±19	90±16	45±15

(mg/dl)	(n = 5)	(n = 8)	(n = 5)	(n = 7)	(n = 9)	(n = 5)
(2w old)						
Blood Glucose	141±22	142±13	140±33	149±41	No data	No data

(mg/dl)	(n = 5)	(n = 5)	(n = 5)	(n = 3)		
(4m old)						

d : vs *Gcg^gfp/gfp^Arx^PL/Y^*, P<0.05. ^ee^: vs *Gcg^gfp/+^Arx^7/Y^*, P<0.01. ^f^: vs *Gcg^gfp/gfp^Arx^7/Y^*, P<0.05. ^ff^: vs *Gcg^gfp/gfp^Arx^7/Y^*, P<0.01.

### Expression of mRNAs for *Gfp*, *glucagon*, *insulin* and *Arx* in the pancreas of *Gcg/Arx* double mutant mice

In *Gcg^gfp/gfp^* mice, hyperplasia of GFP-positive α-like cells results in an increased level of *Gfp* mRNA and an absence of *glucagon* mRNA [3]. To address the effect of impaired ARX function on the pancreas, we analyzed expression of *Gfp*, *glucagon*, *insulin* and *Arx* in the *Gcg/Arx* double mutant mice.

**Figure 3:**
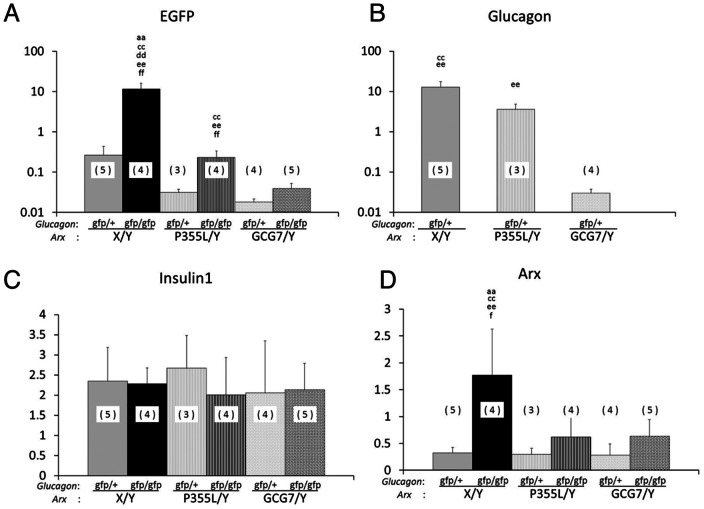
Expression of *Gfp*, *glucagon*, *insulin* and *Arx* mRNAs in the pancreas of *Gcg/Arx* double mutant mice. Relative mRNA levels for *Gfp* (A) *glucagon* (B), *insulin1* (C), and *Arx* (D) in the pancreas of 2 weeks old mice are shown. Number of animals is indicated in parenthesis in each column. ‘aa’ vs *Gcg^gfp/+^Arx^+/Y^*, P<0.01; ‘bb’ vs *Gcg^gfp/gfp^Arx^+/Y^*, P<0.01; ‘cc’ vs *Gcg^gfp/+^Arx^PL/Y^*, P<0.01; ‘dd’ vs *Gcg^gfp/gfp^Arx^PL/Y^*, P<0.01; ‘ee’ vs *Gcg^gfp/+^Arx^7/Y^*, P<0.01; ‘f’ vs *Gcg^gfp/gfp^Arx^7/Y^*, P<0.05; ‘ff’ vs *Gcg^gfp/gfp^Arx^7/Y^*, P<0.01.

As shown in Fig. 3A, the levels of *Gfp* mRNA were significantly higher in the pancreas of *Gcg^gfp/gfp^Arx^+/Y^* mice than *Gcg^gfp/+^Arx^+/Y^* mice; *Gfp* levels were significantly lower in *Gcg^gfp/gfp^Arx^PL/Y^* and *Gcg^gfp/gfp^Arx^7/Y^* mice than in *Gcg^gfp/gfp^Arx^+/Y^* mice. This result indicated that *Gfp* mRNA expression and/or hyperplasia of α-like cells are reduced in the *Arx* mutant mice. *Glucagon* mRNA was detected in *Gcg^gfp/+^*, but not *Gcg^gfp/gfp^* mice, and its expression was also significantly lower in *Arx* mutant mice (Fig. 3B). As expected from the difference in functional impairment of ARX-PL and ARX-7, the decreases in *Gfp* and *glucagon* mRNAs were more evident in *Arx^7/Y^* mice than in *Arx^PL/Y^* mice. The levels of *insulin* mRNA did not differ significantly among the *Gcg/Arx* double mutants, suggesting that ß-cell mass and/or gene expression in ß-cells are marginally affected by *Arx* mutation and by presence or absence of proglucagon-derived peptides (Fig. 3C). The level of *Arx* mRNA was also increased in the *Gcg^gfp/gfp^* mice, and was also lower in *Arx* mutant mice (Fig. 3D).

### Islet area, islet number and pancreas size in the *Gcg/Arx* double mutant mice

**Figure 4:**
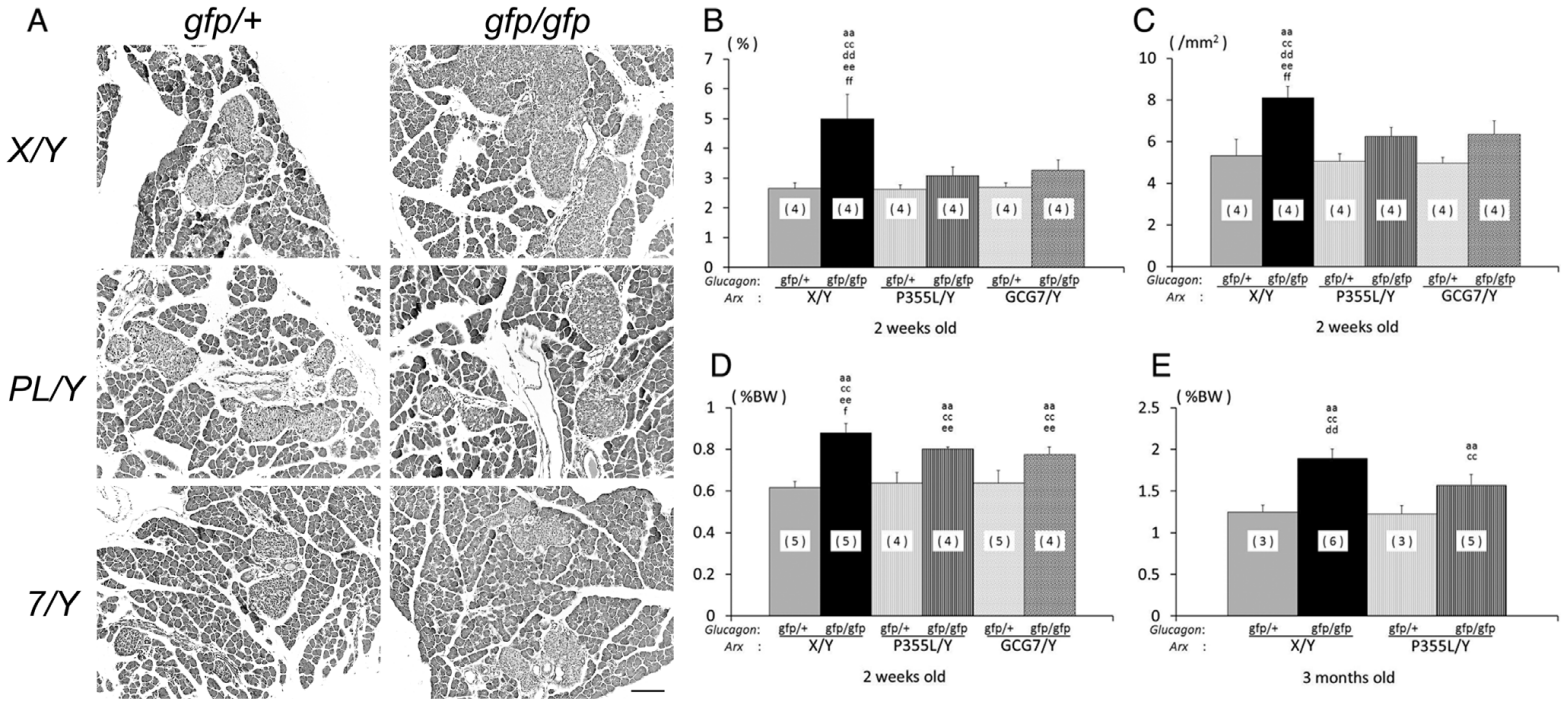
Morphometric analyses of the pancreas in *Gcg/Arx* double mutant mice. (A) Representative HE-stained images of pancreas sections. (B) Relative dimension/area of islets per pancreas in 2-week-old mice. (C) Relative number of islets per mm^2^ in the pancreas of 2-week-old mice. (D) Relative pancreas weight to total body weight in 2-week-old mice. (E) Relative pancreas weight in 3-month-old mice. Number of animals is indicated in parenthesis in each column. ‘aa’ vs *Gcg^gfp/+^Arx^+/Y^*, P<0.01; ‘cc’ vs *Gcg^gfp/+^Arx^PL/Y^*, P<0.01; ‘dd’ vs *Gcg^gfp/gfp^Arx^PL/Y^*, P<0.01; ‘ee’ vs *Gcg^gfp/+^Arx^7/Y^*, P<0.01; ‘ff’ vs *Gcg^gfp/gfp^Arx^7/Y^*, P<0.01.

The number of islets and the area they encompass are significantly increased in the *Gcg^gfp/gfp^* mice [3], [15]. To address whether functionally defective mutation of ARX influence islet number and area, we performed a histological analysis of the pancreas of *Gcg/Arx* double mutant mice at 2 weeks of age. As shown in the representative section Fig. 4A, islet area in the *Gcg^gfp/gfp^Arx^+/Y^* pancreas was greater than in the *Gcg^gfp/+^Arx^+/Y^* pancreas. Morphometric analyses confirmed that both islet area (Fig. 4B) and islet number (Fig. 4C) were increased in *Gcg^gfp/gfp^Arx^+/Y^* mice. However, the increase was significantly lower in *Gcg^gfp/gfp^Arx^PL/Y^* and *Gcg^gfp/gfp^Arx^7/Y^* mice. These results indicate that ARX is involved in the increase in islet number and area caused by the absence of proglucagon-derived peptides.

An increase in pancreas size/weight has been documented in animal models with defects in glucagon action [3], [16], although the mechanism remains unclear. In the present study, we found no evidence of a significant difference in relative pancreas weights among *Gcg^gfp/+^Arx^+/Y^*, *Gcg^gfp/+^Arx^PL/Y^* and *Gcg^gfp/+^Arx^7/Y^* mice. The pancreas weight of *Gcg^gfp/gfp^Arx^PL/Y^* and *Gcg^gfp/gfp^Arx^7/Y^* mice was significantly larger than in *Gcg^gfp/+^*mice (Fig. 4D). However, *Gcg^gfp/gfp^Arx^+/Y^* and *Gcg^gfp/gfp^Arx^7/Y^* mice showed a significant difference in pancreas weight at 2 weeks of age, and *Gcg^gfp/gfp^Arx^+/Y^* and *Gcg^gfp/gfp^Arx^PL/Y^* were significantly different at 3 months of age (Fig. 4E). Three-month-old *Gcg^gfp/gfp^Arx^7/Y^* mice were not available for this analysis because of their shortened life span. Overall, the results suggest that ARX is also involved in the increase in pancreas weight caused by the absence of proglucagon-derived peptides. However, the impact of functionally defective ARX on pancreas weight is not as dramatic as that on islet endocrine cells.

### Immunohistochemical analyses and fluorescent imaging of the *Gcg/Arx* double mutant pancreas

**Figure 5:**
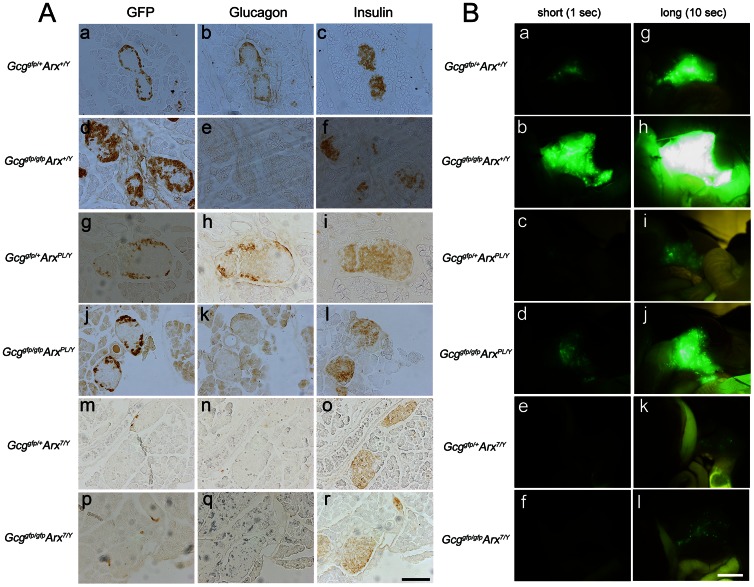
Immunohistochemical analyses and fluorescent imaging of the pancreas in *Gcg/Arx* double mutant mice. (A) Immunohistochemical analysis using anti-GFP (left column: a, d, g, j, m, and p), anti-glucagon (middle column: b, e, h, k, n and q) and anti-insulin (right column: c, f, I, l, o and r) antibodies. Pancreas from 2-week-old mice with the indicated genotypes was analyzed. a–c, *Gcg^gfp/+^Arx^+/Y^*; d–f, *Gcg^gfp/gfp^Arx^+/Y^*; g–i, *Gcg^gfp/+^Arx^PL/Y^*; j–l, *Gcg^gfp/gfp^Arx^PL/Y^*; m–o, *Gcg^gfp/+^Arx^7/Y^*; p–r, *Gcg^gfp/gfp^Arx^7/Y^*. Scale bar, 100 µm. (B) GFP fluorescent images of the pancreas. The 6 images in the left column (a–f) and the 6 images in right (g–l) are identical except for the exposure time. a and g, *Gcg^gfp/+^Arx^+/Y^*; b and h, *Gcg^gfp/gfp^Arx^+/Y^*; c and I, *Gcg^gfp/+^Arx^PL/Y^*; d and j, *Gcg^gfp/gfp^Arx^PL/Y^*; e and k, *Gcg^gfp/+^Arx^7/Y^*; and f and l, *Gcg^gfp/gfp^Arx^7/Y^*. Scale bar, 1 mm.

To characterize the distribution of α/α-like cells and ß-cells in the islets, serial section of the pancreas were immunohistochemically analyzed (Fig. 5A). Immunoreactivity for GFP was markedly increased in the *Gcg^gfp/gfp^Arx^+/Y^* mice compared to *Gcg^gfp/+^Arx^+/Y^* mice (Fig. 5A, a vs d) indicating marked hyperplasia of α-like cells. There is less apparent hyperplasia of α-like cells in the *Gcg^gfp/gfp^Arx^PL/Y^* mice (Fig. 5Aj) and the immunoreactivity was almost comparable to that in *Gcg^gfp/+^Arx^+/Y^* mice (Fig. 5Aa). It was difficult to detect α-cells in *Gcg^gfp/gfp^Arx^7/Y^* mice and α-like cells in *Gcg^gfp/gfp^Arx^7/Y^* mice. Glucagon immunoreactivity was present in the *Gcg^gfp/+^* mice (Fig. 5Ab, h and n), but not in the *Gcg^gfp/gfp^* mice (Fig. 5Ae, k and q), and was difficult to detect in *Gcg^gfp/+^Arx^7/Y^* mice (Fig. 5An). By contrast, immunoreactivity for insulin showed little variation between the different genotypes (Fig. 5Ac, f, i, j, m and r).

As α/α-like cells express GFP in the *Gcg/Arx* double mutant mice, we carried out an *in situ* analysis of the pancreas using an epifluorescent microscope to identify and estimate the numbers of these cells (Fig. 5B). The results were in agreement with those above for the immunohistochemical analyses (Fig. 5A) and the gene expression data (Fig. 2). Taken together, hyperplasia of α-like cells in the absence of proglucagon-derived peptides was reduced in mice with functionally defective ARX. The number of α-cells was also reduced in *Gcg^gfp/+^* mice with functionally defective ARX. The present study on the pancreas and pancreatic islets demonstrated that the ARX-7 mutation has a greater effect than ARX-PL, a conclusion that is in agreement with the results of neurological analyses [10].

## Discussion

Hyperplasia of α-cells has been documented in various mouse models that have defective glucagon action, such those deficient in glucagon receptor [16] or in prohormone convertase 2 that excise glucagon from its precursor proglucagon [17]. Recently, hyperplasia of α-cells has been also shown in mice with a liver-specific knock out of glucagon receptor, suggesting that circulating factors other than glucagon itself regulate α-cell proliferation [18].

The *Gcg^gfp/gfp^* mice lack proglucagon-derived peptides, including glucagon and GLP-1, and, as adults, they are normoglycemic. In these aspects, the *Gcg^gfp/gfp^* mice are a contrast to with the animal models mentioned above, which exhibit lower blood glucose levels and elevated GLP-1 levels. Nevertheless, the *Gcg^gfp/gfp^* mice develop hyperplasia of GFP-positive α-like cells and this result indicates that neither lower blood glucose levels nor elevated GLP-1 levels are prerequisite for the hyperplasia [3], [4]. This conclusion is in agreement with a recent report on glucagon receptor/GLP-1 receptor double knockout mice, which are normoglycemic and develop α-cells hyperplasia [19].

As the underlying mechanisms for hyperplasia of α/α-like cells under defective glucagon action remain largely unknown, we sought to characterize the possible role of ARX in hyperplasia in the present study. We showed that hyperplasia of α-like cells due to absence of the proglucagon-derived peptides was reduced in male mice lacking a fully functional ARX. In particular, the number of α-cells in the *Gcg^gfp/+^* background and α-like cells in the *Gcg^gfp/gfp^* background were considerably lower in the mice which carried only ARX-7. The clinical severity of the condition shown by human patients carrying mutations corresponding to those here and the neurological studies of animal models, demonstrates that functional impairment of ARX-7 is more severe than that of ARX-PL [10]. Therefore, our data indicated that the function of ARX is one of the most important modifiers of the number of islet α/α-like cells.

The present study by itself cannot exclude the possibility that the islet phenotype is secondary to neurological disorder caused by defective ARX function. However, as α-cell specific ablation of ARX causes extensive loss of α-cells [20], it is likely that functional impairment of ARX in the islet endocrine cells plays the major role in the islet phenotype observed in the present study.

In addition to the increase in islet mass, the size of the pancreas was increased in *Gcg^gfp/gfp^* mice [4] and in glucagon receptor deficient mice [16]. The effect on pancreas size was also found to be diminished in the present study (Fig. 4D and E), suggesting that ARX is involved in growth of exocrine glands. Interestingly, both loss of α-cells and morphological abnormalities in exocrine glands have been documented in human patients with *Arx*-null mutation [21]. As ARX is not expressed in exocrine glands, it is suggested that ARX-dependent signals, *i.e.* growth factors, from the endocrine pancreas control development and/or maintenance of the exocrine pancreas.

Hyperplasia of α-cells under defective glucagon signaling is not unique to rodent models, and a human case carrying a homozygous glucagon receptor mutation has been reported to show α-cell hyperplasia and islet cell tumor [22]. Furthermore, proliferation of α-cells and disregulated glucagon production have been recently highlighted in the pathogenesis of diabetes mellitus [23], [24]. The present study showed that ARX plays an important role in the control of α-cell numbers, and suggests that modification of ARX function and/or expression in islets should be considered as a possible target for suppression of α-cell proliferation.
